# Nucleolin as activator of *TCF7L2* in human hematopoietic stem/progenitor cells

**DOI:** 10.1038/s41375-021-01434-8

**Published:** 2021-11-19

**Authors:** Sven Reister, Csaba Mahotka, Edgar Grinstein

**Affiliations:** 1 Department of Pediatric Oncology, Hematology and Clinical Immunology, Medical Faculty, Heinrich Heine University, Düsseldorf, Germany; 2 Institute of Pathology, Medical Faculty, Heinrich Heine University, Düsseldorf, Germany; 3 Department of Hematology, Oncology and Clinical Immunology, Medical Faculty, Heinrich Heine University, Düsseldorf, Germany

**Keywords:** Haematopoietic stem cells, Cell signalling

## To the Editor:

Nucleolin is a multifunctional factor of growing and cancer cells [1, 2]. It is a candidate molecular target for cancer therapy [2], aberrantly active in certain hematological malignancies [1, 3]. Biological processes involving nucleolin include, but are not limited to, gene transcription, chromatin remodeling, RNA metabolism, translation and cell-surface signaling [1–4]. Nucleolin is predominantly expressed in hematopoietic stem/progenitor cells (HSPCs) versus differentiated hematopoietic tissue, amplifies long-term culture-initiating cells and promotes execution of the HSC gene expression program [1, 3, 4]. It also counteracts GSK3β to promote Wnt signaling and facilitates Akt signaling and a cytokine-dependent long-term maintenance of HSPCs [3, 5]. Working out the role of nucleolin in stem cell-governing signaling will improve understanding of the molecular contexts of HSPCs.

Wnt signaling in stem cell control can guide tissue renewal and regeneration and is hijacked in certain leukemia types [6–9]. Here we find that, in human CD34 + HSPCs, nucleolin is associated with the molecular signature regulation of Wnt signaling whose member transcription factor 7-like 2 (TCF7L2), implicated also in regeneration of hematopoietic lineages [7], is partially involved in the transcriptional upregulation of the signature genes. Furthermore, nucleolin is a *TCF7L2* promoter-binding factor that activates *TCF7L2*. The study provides new insights into molecular network relevant to stem/progenitor cells in normal and malignant hematopoiesis and suggests that deregulated nucleolin may favor aberrant Wnt signaling in certain cancers.

**Fig. 1:**
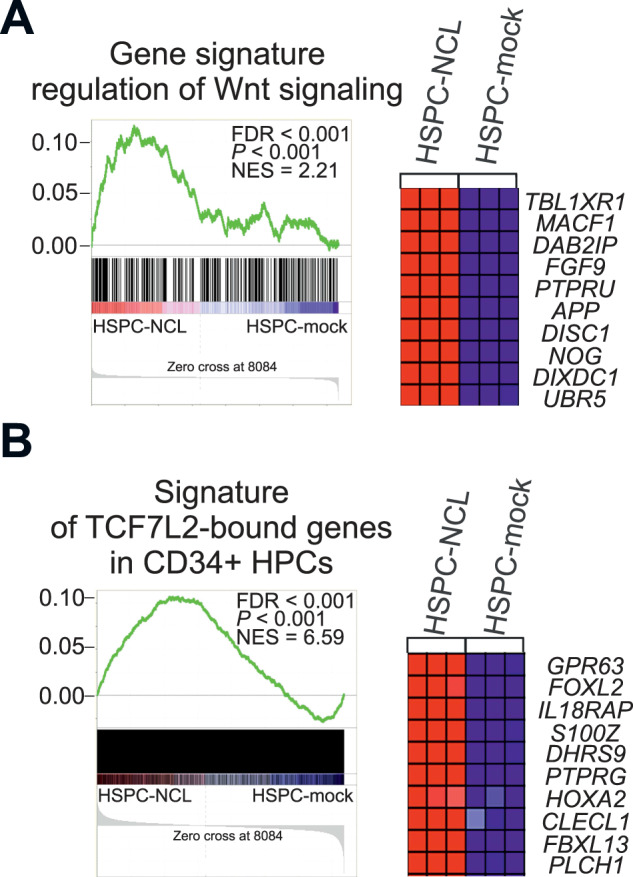
Gene signatures of Wnt signaling regulation. (**A**) and TCF7L2-bound genes [7] (**B**), enriched in human HSPCs by nucleolin. Nucleolin-dependent HSPC-derived expression profile [4] was used for GSEA (Supplementary Methods). Heatmaps depict top 10 genes from leading-edge subsets.

Figure 1A shows that gene signature regulation of Wnt signaling was enriched by nucleolin as was determined by gene set enrichment analysis (GSEA), and transcriptionally upregulated Wnt signaling regulators included *TCF7L2* (Table S1A). GSEA used nucleolin-dependent expression profile from mobilized peripheral blood (MPB) HSPCs [4], that are exploited in hematological transplantology for hematopoietic reconstitution [10]. Involved in certain facets of hematopoiesis, TCF7L2 belongs to the TCF/LEF family of DNA-binding nuclear factors, and its association with N-terminally dephosphorylated (active) β-catenin leads to activation of TCF7L2-bound genes (Supplementary Information, page 4). TCF7L2 and N-terminally dephosphorylated β-catenin were significantly upregulated in HSPCs carrying nucleolin expression vector (HSPC-NCL) versus control HSPCs carrying expression vector of N-terminally truncated nucleolin, amino-acid (aa) residues 289-709 (HSPC-NCL-289-709), or with no cDNA (HSPC-mock) (Fig. S1). In addition, nucleolin was upregulated ~4-fold and a signature of TCF7L2-bound genes, derived from CD34 + MPB HPCs [7], was enriched in HSPC-NCL cells, indicating activation of TCF7L2-associated transcription (Fig. 1B, [4] and data not shown). Moreover, genes bound by TCF7L2 were overrepresented among the genes encoding Wnt signaling regulators upregulated in HSPC-NCL cells (Table S1) (48%, *P* < 0.016), and this included several reported as TCF7L2 downstream-regulated genes (Supplementary Information, page 4). Thus, in CD34 + HSPCs, nucleolin is associated with the molecular signature regulation of Wnt signaling and the transcriptional upregulation of the signature genes partially involves TCF7L2.

**Fig. 2:**
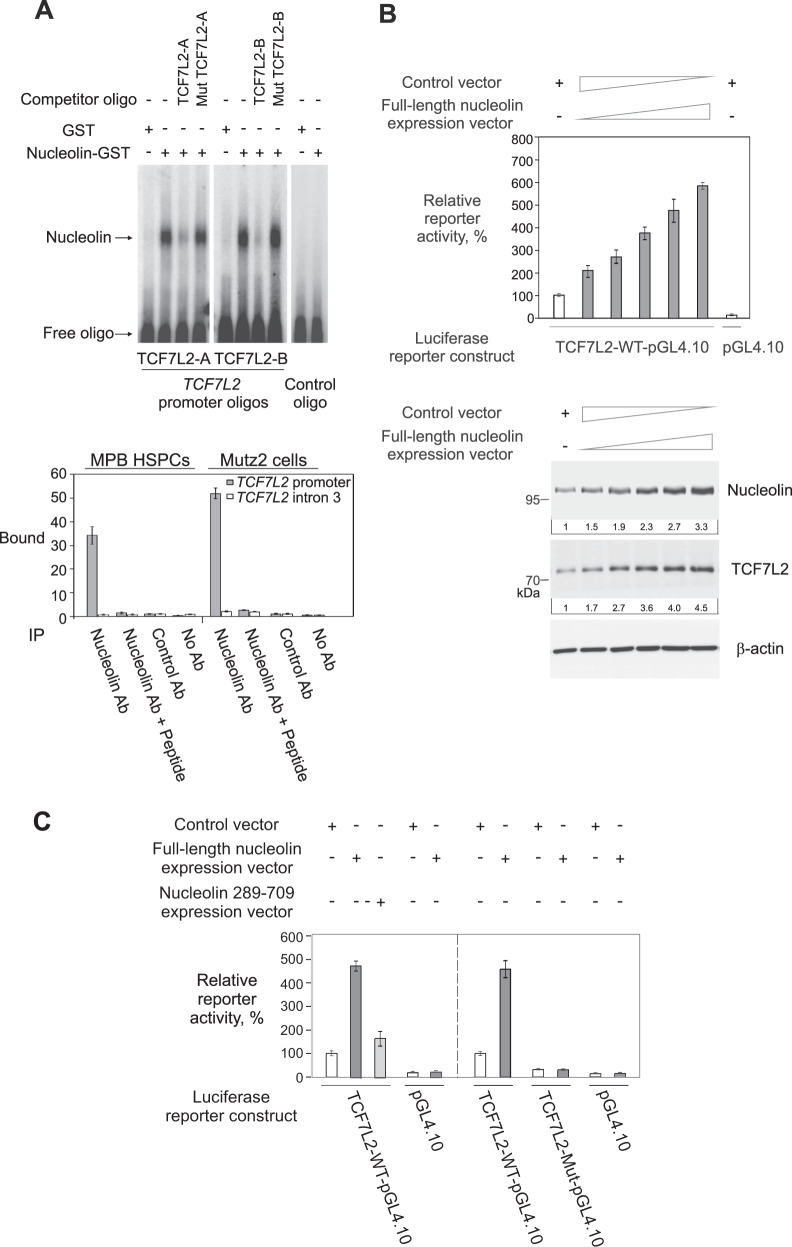
Nucleolin binds to *TCF7L2* promoter and activates *TCF7L2*. **A** Top, EMSA with labeled *TCF7L2* promoter oligonucleotides. Bottom, ChIP of *TCF7L2* promoter (Table S2) with anti-nucleolin antibody. **B** Top, luciferase assay with *TCF7L2* promoter reporter construct co-nucleofected with increasing amounts of nucleolin expression vector. Bottom, immunoblot analysis of nucleolin and TCF7L2 (Table S3). **C** Luciferase assay after co-nucleofection of *TCF7L2* promoter reporter constructs with indicated expression constructs. The means ± SD are shown; *n* = 3. The *y*-axis in (**A**) indicates the ratio between bound and input DNA (arbitrary units) and, in (**B**, **C**), activity relative to *TCF7L2* promoter reporter construct co-nucleofected with empty expression vector.

Furthermore, *TCF7L2* promoter harbored nucleolin binding sites and, in EMSA experiments, affinity-purified nucleolin-GST protein bound directly and sequence-specifically to *TCF7L2* promoter-derived oligonucleotides containing nucleolin recognition motifs (Fig. 2A and Supplementary Methods). In addition, anti-nucleolin antibody co-precipitated efficiently *TCF7L2* promoter in chromatin immunoprecipitation (ChIP) experiments with CD34 + MPB HSPCs and CD34 + CD133 + cells Mutz2, derived from PB of an AML patient (Fig. 2A, Supplementary Methods). Moreover, as measured in Mutz2 cells available in sufficient quantities, nucleolin activated *TCF7L2* promoter reporter construct in a concentration-dependent fashion, and also cellular TCF7L2 levels were nucleolin-modulated after overexpression or knockdown of nucleolin (Fig. 2B and data not shown). Furthermore, truncated nucleolin aa 289-709, devoid of N-terminal domain, only marginally activated wild-type *TCF7L2* promoter, and a promoter derivative lacking sequence motifs required for nucleolin binding was not nucleolin-dependent and largely inactive (Figs. 2C and S2). Thus, nucleolin is a *TCF7L2* promoter-binding factor that activates *TCF7L2*.

Wnt signaling participates in HSPC homeostasis, whereas its deregulation in leukemogenesis is capable of conferring LSC properties [8, 9] (Supplementary Discussion). The effect of nucleolin on Wnt signaling regulators suggests its relevance to regulation of Wnt signaling. Future analysis, involving more purified stem cell phenotype, may further detail its function for Wnt signaling in hematopoiesis. Since nucleolin is implicated in cell transformation [1–3], our findings suggest that its altered activity participates in Wnt signaling deregulation in certain cancers.

This study, mainly focused on the connection between nucleolin and TCF7L2, describes that transcriptional upregulation of Wnt signaling regulators by nucleolin in CD34 + HSPCs in part involves TCF7L2. Furthermore, nucleolin interacts with the *TCF7L2* promoter to activate *TCF7L2*. The study provides new insights into molecular network relevant to stem/progenitor cells in normal and neoplastic hematopoiesis and suggests that deregulated nucleolin may favor aberrant Wnt signaling in certain cancers. Future research will also further examine its role in renewal and regeneration of hematopoietic tissues.

## Supplementary information

Supplemental material
