# Supplementary material for: Nucleolin as activator of TCF7L2 in human hematopoietic stem/progenitor cells
Source: Leukemia. 2021 Nov 19;35(12):3616–8. doi: 10.1038/s41375-021-01434-8 (PMC8632675; doi:10.1038/s41375-021-01434-8)

Supplementary information

Nucleolin as activator of *TCF7L2* in human hematopoietic stem/ progenitor cells

Sven Reister, Csaba Mahotka and Edgar Grinstein

Correspondence: Edgar Grinstein, Ph.D.

E-mail: Edgar.Grinstein@uni-duesseldorf.de

Supplementary Materials and Methods

Cells and culture conditions

MPB HSPCs from patients with non-hematological malignancies collected, CD34-immunopurified and frozen for subsequent use in autologous HSC transplantation, were described.[3] Aliquots of cryopreserved HSPCs from deceased patients designated to be discarded were utilized after informed consent of legal guardians and in accordance with the Ethics Committee of the Medical Faculty of the Heinrich Heine University of Düsseldorf, as described.[3,4] CD34+ cell purity was >90%. In Figure S1, aliquots of lysates from previously described cells were used, transduced with lentivirus encoding for full-length human nucleolin (HSPC-NCL), or with lentivirus encoding for N-terminally truncated nucleolin aa 289-709 (HSPC-NCL-289-709), or with lentivirus devoid of nucleolin cDNA (HSPC-mock).[4] The lysates were from cell populations containing ~90% pure transduced viable cells.

In experiments where quantities of HSPCs were not sufficient, CD34+CD133+ cell line Mutz2, derived from PB of an acute myelogenous leukemia (AML) patient,[S1] was used as described.[3] Mutz2 cells (ACC-271, DSMZ, Braunschweig, Germany) were cultured in α-MEM

supplemented with 20% FCS and 20% conditioned medium from 5637 cells (ACC-35, DSMZ). Cells were recently authenticated and tested for mycoplasma contamination.

Electrophoretic mobility shift assay (EMSA) and ChIP assay

TCF7L2 gene promoter upstream of the translation start site was described in the previous study.[S4] A search of this promoter (GenBank accession number AF522996) for potential nucleolin binding sites revealed the presence of two fragments that included the following sequences: nucleotides (nt) -1215 to -1208 (5'-TGAAATGA-3') and nt -427 to -420 (5'-TGAACTGA-3'). Double-stranded oligonucleotides from the *TCF7L2* promoter used in EMSA contained nt -1232 to -1193 of the *TCF7L2* promoter (TCF7L2-A) or nt -440 to -406 (TCF7L2-B). The respective derivatives of the oligonucleotides TCF7L2-A and TCF7L2-B were devoid of nt -1213 to -1209 (Mut TCF7L2-A), or nt -425 to -421 (Mut TCF7L2-B). Double-stranded oligonucleotide carrying two copies of the sequence 5'-CCCTTTGATCTTACC-3' that contains the optimal TCF binding motif[S5] was used as control oligonucleotide. Nucleolin-glutathione S-transferase (GST) fusion protein, comprising amino-acid residues 289-709 of nucleolin, and use thereof in EMSA was described previously.[S2,S3]

ChIP experiments with protein A/G MicroBeads (Miltenyi Biotec, Gladbach, Germany) followed a protocol provided by Miltenyi Biotec, as described.[3] Antibody specific for N-terminal peptide of nucleolin,[S2,S3] affinity-purified on the peptide column, was used and controls included presaturation of this antibody with the blocking peptide. Immunoprecipitated DNA was quantified by real-time PCR. Primer sequences are summarized in Table S2.

Western blotting

Immunoblot analysis followed standard procedures, and normalized band intensity is shown as a percentage of cells nucleofected with empty expression vector (Figures 2B and S2) or of the HSPC-mock sample (Figure S1). Antibodies, validated previously, were as follows: antibody specific for N-terminal peptide of nucleolin,[S2,S3] purified on the peptide column, and commercially available antibodies described in Table S3.

Luciferase reporter assays

The following *TCF7L2* promoter reporter constructs, cloned into pGL4.10 reporter vector (Promega, Madison, WI, USA), were used: TCF7L2-WT-pGL4.10 construct, containing the full-length wild-type human *TCF7L2* promoter[S4] (see also Supplementary Methods, page 2), and a derivative lacking nt -1213 to -1209 and -425 to -421 (TCF7L2-Mut-pGL4.10). CMV promoter-driven expression constructs contained the following: i) cDNA encoding for N-terminally FLAG-tagged full-length human nucleolin or ii) cDNA encoding for FLAG-tagged nucleolin amino-acid residues 289-709. Mutz2 cells were nucleofected with Nucleofector Solution-V (Lonza, Cologne, Germany) and luciferase measurements were performed as described.[S3] In Figure 2B, the cells were co-nucleofected with increasing amounts of full-length nucleolin expression vector (none, 0.2, 0.4, 0.6, 0.8 and 1.0 µg) together with the TCF7L2-WT-pGL4.10 reporter construct, and cell aliquots were used for analyzes shown.

Gene set enrichment analysis (GSEA)

GSEA was performed using GSEA v2.2 software (Broad Institute, Cambridge, MA, USA),[S6] and statistical difference was determined by 1000 gene set permutations, timestamp seed for permutation. Minimum gene set size was set to 15; max_probe was used to collapse multiple probe sets / gene. The nucleolin-dependent gene expression profile, comprising data obtained with MPB HSPCs from 3 patients (HSPC-NCL cells versus HSPC-mock control cells), was described and the necessary controls, including the validation of differential gene expression by qRT-PCR, were reported.[4] Nucleolin protein levels were ~4-fold higher in HSPC-NCL versus HSPC-mock cells.[4] Other gene sets used for GSEAs were as follows: in Figure 1A, gene ontology gene set regulation of Wnt signaling pathway and, in Figure 1B, TCF7L2-bound gene set, derived from human CD34+ MPB HPCs.[7]

Supplementary Discussion

Wnt signals are relevant to regulation of HSPCs and the strength of Wnt signaling regulates hematopoiesis.[8,9,S7] On the other hand, deregulated Wnt signaling, linked to worse clinical

outcome for a subset of human cancers,[S8] is causatively associated with leukemogenesis and is of importance to the properties of LSCs.[8,9,S9,S10,S11] The effect of nucleolin on Wnt signaling regulators including *TCF7L2* suggests its relevance to regulation of Wnt signaling.

TCF7L2 (also known as TCF4) is upregulated in HSCs/HSPCs[S12] and is capable of enhancing GATA2-mediated transcriptional activation.[7] Furthermore, TCF7L2 is involved in hematopoietic regeneration and differentiation and cooperates with lineage master regulators to affect expression of key hematopoietic genes.[7] On the other hand, its expression is elevated in CD34+ hematopoietic cells from patients in chronic myeloid leukemia (CML) blast crisis and TCF7L2 has a role in transcriptional changes in CML.[S13] In addition, overexpression of TCF7L2 in mantle cell lymphoma-initiating cells is associated with aberrant Wnt activity critical for their maintenance and survival.[S14] Additionally, installation of a cancer-promoting Wnt/SIX1 signaling axis by the MLL-AF9 oncoprotein in AML LSCs involves TCF7L2.[S15] TCF7L2 participates as well in aberrant Wnt activity in certain non-hematological malignancies.[S5,S16-S18]

TCF7L2 is a member of the TCF/LEF family of DNA-binding nuclear factors that are effectors of the Wnt signaling pathway, and interaction of β-catenin with TCF7L2 leads to activation of TCF7L2-bound genes.[S5,S17,S19] In addition, this interaction also serves as a target for cancer therapy.[S14,S17,S18,S20] The herein-reported finding that nucleolin, as a *TCF7L2* promoter-binding factor, upregulates *TCF7L2* is novel and, in line with our published data,[3,5] nucleolin as well elevates the level of N-terminally dephosphorylated β-catenin that, as was demonstrated by Staal and colleagues,[S21] transduces Wnt signals. Thereby, the signature of TCF7L2-bound genes is enriched by nucleolin (Figure 1B). Furthermore, genes bound by TCF7L2 are overrepresented among the upregulated genes encoding regulators of Wnt signaling. This includes *BAMBI*, *LRP4*, *MDFIC* and *SOX4*, that were shown to be TCF7L2-occupied[S22,S23] and modulatable via TCF7L2[S18,S24-S27] and β-catenin[S18,S24,S28-S30] in non-HSPCs. Thus, transcriptional upregulation of Wnt signaling regulators by nucleolin partially involves TCF7L2.

Acknowledgments

The work was funded, PI E. Grinstein, by DFG, grant GR 3581/2-1, José Carreras Leukämie-Stiftung, grant DJCLS R 12/32, and the Forschungskommission of the Medical Faculty of the Heinrich Heine University of Düsseldorf. We thank Prof. Ulrich Germing and the former director of the clinic, Prof. Rainer Haas, for kind support. We also thank Prof. Elmar Gren for helpful discussions.

Supplementary References

S1 Kratz-Albers K, Zuhlsdorp M, Leo R, Berdel WL, Buchner T, Serve H. Expression of AC133, a novel stem cell marker, on human leukemic blasts lacking CD34-antigen and on a human CD34^{+} leukemic line: MUTZ-2. *Blood* 1998; **92**: 4485-4487.

S2 Grinstein E, Wernet P, Snijders PJ, Rösl F, Weinert I, Jia W *et al*. Nucleolin as activator of human papillomavirus type 18 oncogene transcription in cervical cancer. *J Exp Med* 2002; **196**: 1067-1078.

S3 Grinstein E, Du Y, Santourlidis S, Christ J, Uhrberg M, Wernet P. Nucleolin regulates gene expression in CD34 positive hematopoietic cells. *J Biol Chem* 2007; **282**: 12439-12449.

S4 Rother K, Johne C, Spiesbach K, Haugwitz U, Tschöp K, Wasner M *et al*. Identification of Tcf-4 as a transcriptional target of p53 signalling. *Oncogene* 2004; **23**: 3376–3384.

S5 Korinek V, Barker N, Morin P, van Wichen D, de Weger R, Kinzler K *et al*. Constitutive Transcriptional Activation by a β-Catenin–Tcf Complex in APC-/- Colon Carcinoma. *Science* 1997; **275**: 1784–1787.

S6 Subramanian A, Tamayo P, Mootha V, Mukherjee S, Ebert B, Gillette M *et al*. Gene set

enrichment analysis: A knowledge-based approach for interpreting genome-wide expression profiles. *Proc Natl Acad Sci USA* 2005; **102**: 15545-15550.

S7 Luis TC, Naber B, Roozen P, Brugman M, de Haas E, Ghazvini M *et al*. Canonical Wnt Signaling Regulates Hematopoiesis in a Dosage-Dependent Fashion. *Cell Stem Cell* 2011; **9**: 345–356.

S8 Zhan T, Rindtorff N, Boutros M. Wnt signaling in cancer. *Oncogene* 2017; **36**: 1461–1473.

S9 Gang E, Hsieh Y, Pham J, Zhao Y, Nguyen C, Huantes S *et al*. Small-molecule inhibition of CBP/catenin interactions eliminates drug-resistant clones in acute lymphoblastic leukemia. *Oncogene* 2014; **33**: 2169–2178.

S10 Staal FJT, Famili F, Perez LG, Pike-Overzet K. Aberrant Wnt Signaling in Leukemia. *Cancers* 2016; **8**: 78.

S11 Ruan Y, Kim H, Ogana H, Kim YM. Wnt Signaling in Leukemia and Its Bone Marrow Microenvironment. *Int J Mol Sci* 2020; **21**: 6247.

S12 Novershtern N, Subramanian A, Lawton LN, Mak RH, Haining N, McConkey ME *et al*. Densely interconnected transcriptional circuits control cell states in human hematopoiesis. *Cell* 2011; **144**: 296–309.

S13 Desterke C, Hugues P, Hwang J, Bennaceur-Griscelli A, Turhan G. Embryonic Program Activated during Blast Crisis of Chronic Myelogenous Leukemia (CML) Implicates a TCF7L2 and MYC Cooperative Chromatin Binding. *Int J Mol Sci* 2020; **21**: 4057.

S14 Mathur R, Sehgal L, Braun F, Berkova Z, Romaguerra J, Wang M *et al*. Targeting Wnt pathway in mantle cell lymphoma-initiating cells. *J Hematol Oncol* 2015; **8**: 63.

S15 Zhang L, Kang X, Lu J, Zhang Y, Wu X, Wu G *et al*. Installation of a cancer promoting WNT/SIX1 signaling axis by the oncofusion protein MLL-AF9. *EBioMedicine* 2019; **39**: 145–158.

S16 Sánchez-Tillóa E, de Barrios O, Siles L, Cuatrecasas M, Castells A, Postigo A. β-catenin/TCF4 complex induces the epithelial-to-mesenchymal transition (EMT)-activator ZEB1 to regulate tumor invasiveness. *Proc Natl Acad Sci USA* 2011; **108**: 19204–19209.

S17 Nusse R, Clevers H. Wnt/β-Catenin Signaling, Disease, and Emerging Therapeutic Modalities. *Cell* 2017; **169**: 985-999.

S18 Fang L, Zhu Q, Neuenschwander M, Specker E, Wulf-Goldenberg A, Weis W *et al*. Small-Molecule Antagonist of the β-Catenin/TCF4 Interaction Blocks the Self-Renewal of Cancer Stem Cells and Suppresses Tumorigenesis. *Cancer Res* 2016; **76**: 891-901.

S19 MacDonald B, Tamai K, He X. Wnt/β-catenin signaling: components, mechanisms, and diseases. *Dev Cell* 2009; **17**: 9–26.

S20 Zhang Y, Wang X. Targeting the Wnt/β-catenin signaling pathway in cancer. *J Hematol Oncol* 2020; **13**: 165.

S21 Staal FJ, van Noort M, Strous GJ, Clevers HC. Wnt signals are transmitted through N-terminally dephosphorylated β-catenin. *EMBO Rep* 2002; **3**: 63-68.

S22 Hatzis P, van der Flier L, van Driel M, Guryev V, Nielsen F, Denissov S *et al*. Genome-Wide Pattern of TCF7L2/TCF4 Chromatin Occupancy in Colorectal Cancer Cells. *Mol Cell Biol* 2008; **28**: 2732-2744.

S23 Zhang J, Zhang J, Yan W, Wang Y, Han L, Yue X *et al*. Unique genome-wide map of TCF4 and STAT3 targets using ChIP-seq reveals their association with new molecular subtypes of Glioblastoma. *Neuro-oncology* 2013; **15**: 279–289.

S24 Sekiya T, Adachi S, Kohu K, Yamada T, Higuchi O, Furukawa Y *et al*. Identification of BMP and Activin Membrane-bound Inhibitor (*BAMBI*), an Inhibitor of Transforming Growth Factor-β Signaling, as a Target of the β-Catenin Pathway in Colorectal Tumor Cells. *J Biol Chem* 2004; **279**: 6840-6846.

S25 Wenzel J, Rose K, Haghighi E, Lamprecht G, Rauen G, Freihen V *et al*. Loss of the nuclear Wnt pathway effector TCF7L2 promotes migration and invasion of human colorectal cancer cells. *Oncogene* 2020; **39**: 3893-3909.

S26 Zhou Y, Park S, Su J, Bailey K, Ottosson-Laakso E, Shcherbina L *et al*. TCF7L2 is a master regulator of insulin production and processing. *Hum Mol Genet* 2014; **23**: 6419-6431.

S27 Van der Flier L, Sabates-Bellver J, Oving I, Haegebarth A, De Palo M, Anti M *et al.* The Intestinal Wnt/TCF Signature. *Gastroenterology* 2007 **132**: 628-632.

S28 Mahmoudi T, Boj S, Hatzis P, Li V, Taouatas N, Vries R *et al*. The Leukemia-Associated Mllt10/Af10-Dot1l Are Tcf4/β-Catenin Coactivators Essential for Intestinal Homeostasis. PLoS Biol 2010; **8**: e1000539.

S29 Bridgewater D, Di Giovanni V, Cain J, Cox B, Jakobson M, Sainio K *et al*. β-Catenin Causes Renal Dysplasia via Upregulation of *Tgfβ2* and *Dkk1. J Am Soc Nephrol* 2011; **22**: 718–731.

S30 Melnik S, Dvornikov D, Müller-Decker K, Depner S, Stannek P, Meister M *et al*. Cancer cell specific inhibition of Wnt/β-catenin signaling by forced intracellular acidification. *Cell Discov* 2018; **4**: 37.

Supplement Table Legends

Supplementary Table S1. (A) Genes from the gene ontology gene set regulation of Wnt signaling pathway, upregulated by nucleolin in CD34+ MPB HSPCs. **(B)** Genes from the gene ontology gene set regulation of Wnt signaling pathway, downregulated by nucleolin in CD34+ MPB HSPCs. Nucleolin-dependent expression profile that comprised data obtained with HSPCs from 3 patients was described and the necessary controls, including the validation of differential gene expression by qRT-PCR, were reported.[4] Criteria for listing differentially expressed genes in the Table are as follows: adjusted p-value $\leq$0.01 and fold change cutoff ≥2. Genes are ranked by adjusted p-value. Genes that are bound by TCF7L2, within 4 kilobases upstream or downstream of gene body, in human CD34+ MPB HPCs[7] are indicated.

Supplementary Table S2. Sequences of primers used.

Supplementary Table S3. Antibodies used for immunoblotting.

Supplement Figure Legends

Supplementary Figure S1. Immunoblot analysis of lysates from HSPC-NCL, HSPC-NCL-289-709 and HSPC-mock cells. Top, analysis of the levels of TCF7L2. Bottom, analysis of the levels of N-terminally dephosphorylated β-catenin,[S21] n=3. Nucleolin protein levels were

monitored by immunoblotting and were ~4-fold higher in HSPC-NCL, versus HSPC-NCL-289-709 and HSPC-mock cells (not shown), see also reference 4.

Supplementary Figure S2. After the measurement of the activity of *TCF7L2* promoter, aliquots of samples from Figure 2C, left were used for immunoblot analysis of the levels of nucleolin and FLAG-tagged nucleolin.

Supplementary Tables

Supplementary Table S1A

Gene symbol	Entrez gene ID	P-value	Adjusted p-value	Fold change (upregulated)	Bound by TCF7L2
TBL1XR1	79718	2.2e-07	4.4e-05	2.6	+
MACF1	23499	2.4e-07	4.5e-05	2.3	+
DAB2IP	153090	2.7e-07	4.8e-05	2.6	+
FGF9	2254	4.0e-07	5.6e-05	17.1	+
APP	351	6.9e-07	7.1e-05	4.0	+
PTPRU	10076	7.1e-07	7.1e-05	8.5	-
DISC1	27185	1.5e-06	9.9e-05	2.4	+
NOG	9241	2.6e-06	1.3e-04	90.0	-
DIXDC1	85458	3.6e-06	1.4e-04	5.1	+
UBR5	51366	5.1e-06	1.7e-04	2.8	+
MAPK14	1432	8.9e-06	2.3e-04	2.2	+
CSNK1E	1454	1.6e-05	3.0e-04	2.7	-
RECK	8434	1.7e-05	3.1e-04	4.8	-
SOX4	6659	1.8e-05	3.2e-04	3.1	+
STK3	6788	2.3e-05	3.7e-04	3.3	+
TNKS2	80351	2.2e-05	3.7e-04	2.5	+
RNF220	55182	2.5e-05	3.9e-04	2.2	-
LRRK1	79705	2.8e-05	4.2e-04	2.3	-
TNKS	8658	2.9e-05	4.3e-04	2.4	+
LGR4	55366	4.0e-05	5.1e-04	4.3	+
FZD7	8324	4.6e-05	5.5e-04	4.9	-
LRP4	4038	5.5e-05	6.2e-04	2.7	+
TERT	7015	5.9e-05	6.4e-04	7.6	-
NPHP3	27031	7.2e-05	7.2e-04	2.0	-
CCNY	219771	8.4e-05	7.9e-04	3.2	+
FOXO1	2308	8.7e-05	8.0e-04	2.3	+
ZBED3	84327	1.4e-04	1.1e-03	7.1	-
CDK14	5218	1.4e-04	1.1e-03	3.1	-
BAMBI	25805	1.6e-04	1.2e-03	8.9	+
TLR2	7097	2.1e-04	1.4e-03	8.7	-
VPS35	55737	2.4e-04	1.5e-03	2.2	-
SMURF2	64750	2.7e-04	1.6e-03	3.2	-
IBP2	3485	2.8e-04	1.6e-03	5.7	-
AMER1	139285	4.6e-04	2.3e-03	5.3	-

TCF7L2	6934	4.7e-04	2.4e-03	2.8	+
CITED1	4435	5.8e-04	2.7e-03	3.0	-
INVS	27130	7.5e-04	3.3e-03	3.0	-
MDFIC	29969	7.9e-04	3.4e-03	2.2	+
IFT20	90410	8.1e-04	3.4e-03	2.3	-
RBPJ	3516	1.2e-03	4.5e-03	2.0	+
TMEM88	92162	1.5e-03	6.3e-03	2.1	+
MDFI	4188	2.8e-03	8.6e-03	3.8	-

Supplementary Table S1B

Gene symbol	Entrez gene ID	P-value	Adjusted p-value	Fold change (downregulated)	Bound by TCF7L2
APOE	348	2.7e-07	4.8e-05	10.8	-
LRP1	4035	1.1e-06	8.7e-05	4.8	-
EGR1	1958	1.3e-06	9.3e-05	13.2	-
CTNND1	51588	1.7e-06	1.0e-04	4.3	+
FUZ	80199	9.2e-06	2.3e-04	3.1	-
SRC	6714	1.6e-05	3.1e-04	5.7	-
TLE1	7088	4.8e-05	5.7e-04	2.9	+
KANK1	23189	1.4e-04	1.0e-03	2.0	+
NFATC4	4776	1.8e-04	1.3e-03	4.6	-
CAPRIN2	65981	2.4e-04	1.5e-03	2.0	-
TIAM1	7074	3.3e-04	1.8e-03	2.2	+
IGFBP4	3487	5.6e-04	2.7e-03	3.1	-
SOX17	64321	1.8e-03	6.0e-03	2.1	+
WNT5B	81029	1.8e-03	6.2e-03	6.4	-

Supplementary Table S2

**Amplified gene region**	**Forward primer sequence (5'-3')**	**Reverse primer sequence (5'-3')**
TCF7L2 promoter	CGATCCCCCTTTTTCTATCTGTCAATC	AGCCGAAGATACAGGAGGTG
TCF7L2 intron 3	AATTTTGGCGCCTTGGGATG	ATTCTTGGTTTGTGTGCAGCC

Supplementary Table S3

Antibody	Vendor	Catalogue number
anti-TCF7L2	Santa Cruz, Dallas, TX, USA	sc-271288
anti-N-terminally dephosphorylated, active β-catenin	Merck Millipore, Billerica, MA, USA	05-665
anti-FLAG	Sigma-Aldrich, St. Louis, MO, USA	A8592
anti-β-actin	Sigma-Aldrich, St. Louis, MO, USA	A1978

Supplementary Figures

Supplementary Figure S1

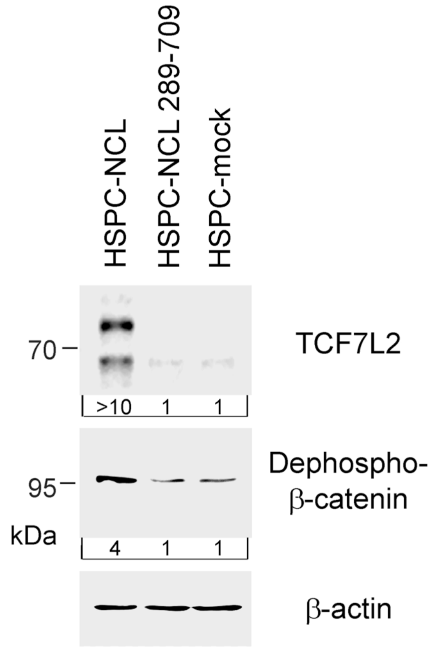

Supplementary Figure S2

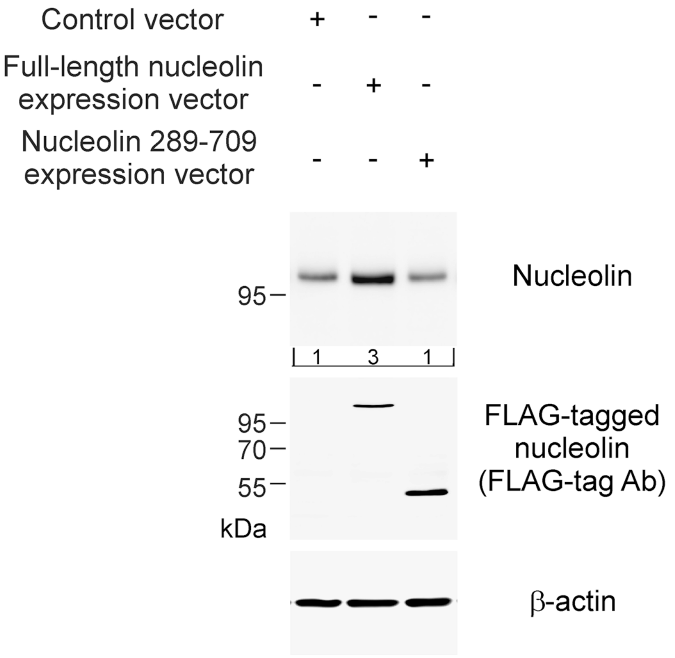

Supplement: Supplementary file 1 — Supplemental material [file 41375_2021_1434_MOESM1_ESM.pdf]
